# Autoreactivity profiles of influenza hemagglutinin broadly neutralizing antibodies

**DOI:** 10.1038/s41598-019-40175-8

**Published:** 2019-03-05

**Authors:** Goran Bajic, Cees E. van der Poel, Masayuki Kuraoka, Aaron G. Schmidt, Michael C. Carroll, Garnett Kelsoe, Stephen C. Harrison

**Affiliations:** 1 Laboratory of Molecular Medicine, Boston Children’s Hospital, Harvard Medical School, Boston, Massachusetts 02115 USA; 2 Program in Cellular and Molecular Medicine, Boston Children’s Hospital, Harvard Medical School, Boston, Massachusetts 02115 USA; 3 Department of Immunology, Duke University, Durham, North Carolina 27710 USA; 4 Department of Microbiology, Harvard Medical School, Boston, Massachusetts 02115 USA; 5 Ragon Institute of MGH, MIT and Harvard, Cambridge, Massachusetts 02139 USA; 6 Duke Human Vaccine Institute, Duke University, Durham, North Carolina 27710 USA; 7 Howard Hughes Medical Institute, Boston, Massachusetts 02115 USA

## Abstract

Epitope-focused approaches for selective clonal induction of broadly neutralizing antibodies (bnAbs) inform most current vaccine strategies for influenza virus and other rapidly evolving pathogens. The two conserved epitopes on the influenza hemagglutinin (HA) - the “stem” and the receptor-binding site (RBS) on the “head” - are the focus of the current “universal” influenza vaccine development efforts. Because stem-directed serum bnAbs are much less abundant than head-directed ones, we hypothesized that the HA stem bnAbs may be autoreactive and thus eliminated through the mechanisms of self-tolerance. We compared autoreactivity profiles of a set of stem and head-directed bnAbs. Most of the stem bnAbs we examined bound autoantigens; several showed staining of HEp-2 cells. A smaller proportion of the head-directed bnAbs were polyreactive. Gene usage did not correlate with autoreactivity. We suggest that complex foreign antigens may often have surface patches resembling some host epitope; our results indicate that HA stem epitopes resemble a host epitope more frequently than does the RBS.

## Introduction

**Figure 1:**
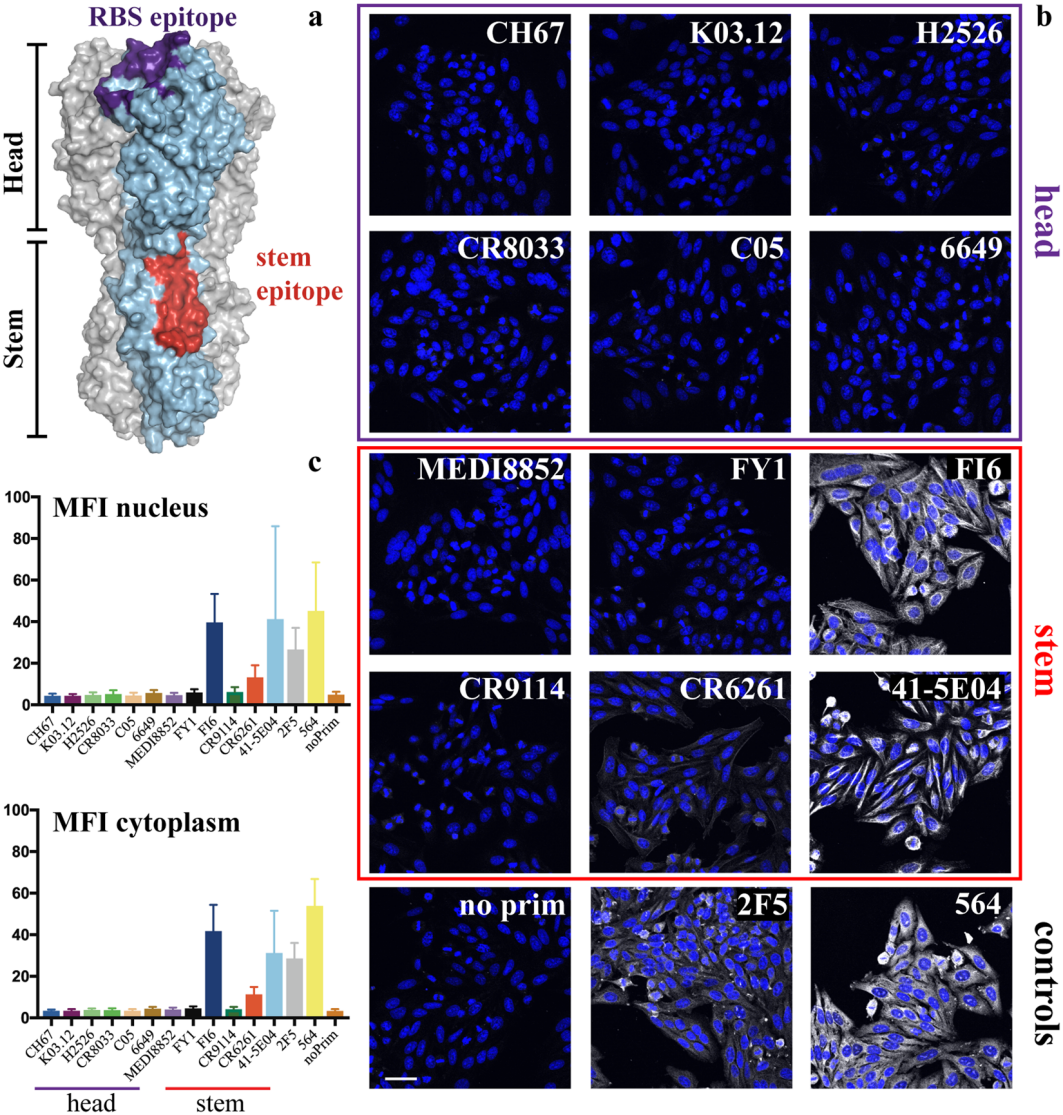
Influenza hemagglutinin (HA) epitopes for broadly neutralizing antibodies and antibody reactivity with the human HEp-2 epithelial cells. (**a**) Atomic model of the influenza hemagglutinin protein. Footprints of two broadly neutralizing antibodies are shown in color: the footprint of RBS-directed CH67 antibody (purple) and that of stem-directed CR6261 (red). The model was derived from the crystal structure of full-length HA (gray and light blue) in complex with mAb CH65 (PDB ID 5UGY), onto which were superposed the HA head complex with mAb CH67 (purple; PDB ID 4HKX) and an HA bound with mAb CR6261 (red; PDB ID 3GBM). HA residues in contact with each antibody are shown in their respective color. Fabs were removed for clarity. (**b**) Representative confocal fluorescence microscopy images of HEp-2 cell staining. Antibody names are indicated for each image. No primary antibody control – no prim and anti-HIV-1 MPER mAb 2F5 were used as controls. All panels are a single plane taken with 20x objective N.A. = 0.7. The scale bar is 50 μm. All HEp-2 cell slides were co-stained with DAPI (blue) to localize the cell nucleus. Channel intensity was adjusted to facilitate visualization of the pattern. Antibodies were grouped and boxed according to their HA epitope – head (purple) and stem (red). (**c**) Mean Fluorescence Intensity (MFI) quantifying nuclear and cytoplasmic signal for each antibody tested. The values are shown as mean ± SD.

Successful viral vaccines, such as those for polio or yellow fever, confer long-lasting immunity by priming the immune system to recognize and neutralize the virus. Some viruses, such as influenza and HIV, evade host immune responses through rapid mutation of surface glycoproteins, thereby changing antigenicity and circumventing previously elicited humoral immunity. The response to current influenza vaccines is often effective only against closely matched strains. Identification of broadly neutralizing antibodies (bnAbs) that recognize diverse influenza viruses has suggested the possibility of “universal” influenza vaccines. The viral hemagglutinin (HA), which binds the host cellular receptor sialic acid and mediates viral entry, is the principal target of known bnAbs^1^. Two conserved regions on HA recognized by bnAbs include the receptor binding site (RBS) on the HA “head” and the membrane-proximal “stem” (Fig. 1a).

RBS-directed bnAbs such as CH67, K03.12 and C05 have sialic acid-like contacts^2,3^ and may be more common than previously thought^4,5^. Antibodies targeting the HA stem are generally rarer. Many derive from V(D)J recombinations with the heavy-chain variable-domain gene segment V_H_1~69^6^. Unlike the RBS-directed antibodies that offer protection through viral neutralization, the primary mechanism of protection of stem-targeting antibodies, in mouse challenge studies, is through FcγR-dependent effector processes such as ADCC^7–11^.

Analogous classes of HIV bnAbs are those whose members recognize the gp120 CD4-binding site and the gp41 membrane proximal external region (MPER), respectively^12,13^. The majority of MPER-directed antibodies bind autoantigens^14–16^, and bnAbs recognizing other epitopes tend likewise to be polyreactive. Deletion by immune tolerance mechanisms might therefore account for the low frequency of antibodies of this kind^14^.

Does breadth of influenza virus neutralization likewise correlate with autoreactivity? The V_H_1~69 gene segment, which encodes over two thirds of known HA stem-directed antibodies^6^, is associated with polyreactive responses in autoimmune pathologies such as Sjögren’s syndrome^17^ and with certain B-cell cancers^18^. It has been suggested that V_H_1~69 antibodies are especially appropriate for stem recognition because they provide a ready-made, hydrophobic contact surface, including an important contact from HCDR2 residue Phe54 seen in various crystal structures^9,19,20^. A previous study^21^ showed that stem-directed antibodies bound more tightly to dsDNA, LPS and insulin than did head binders. V_H_1~69 encoded, anti-HA antibodies that did not bind the stem had much lower affinity for these potential autoantigens, suggesting a correlation between properties that give rise to polyreactivity (e.g., hydrophobicity of the CDR surface and positive charge on HCDR3) and adequate access to stem surfaces occluded on virions. Specific epitopes within the stem and head were not determined. Few, if any, of the antibodies in that panel gave nuclear staining of HEp-2 cells, leading the authors to conclude that the extent of polyreactivity exhibited by stem-directed antibodies might not be pathogenic.

These observations led us to pose two sets of questions. First, for stem-directed antibodies, what are the polyreactivity characteristics of those not encoded by V_H_1~69? Second, like many non-MPER directed HIV bnAbs, do RBS-directed influenza HA bnAbs also tend to show polyreactivity? The broader question is, with what probability does *any* foreign antigen have a B-cell epitope (somewhere on that antigen) similar to an epitope on *some* host protein.

To examine the polyreactivity of HA-directed antibodies, we chose a 12-antibody panel, six specific for the HA stem and six for the head. Of the latter, five bound the RBS and one a non-RBS head epitope. Ten of the antibodies came directly from donor BCR sequences; one of the stem antibodies was generated through *in vitro* affinity maturation, and one of the head antibodies came from screening a human-antibody phage display library.

**Figure 2:**
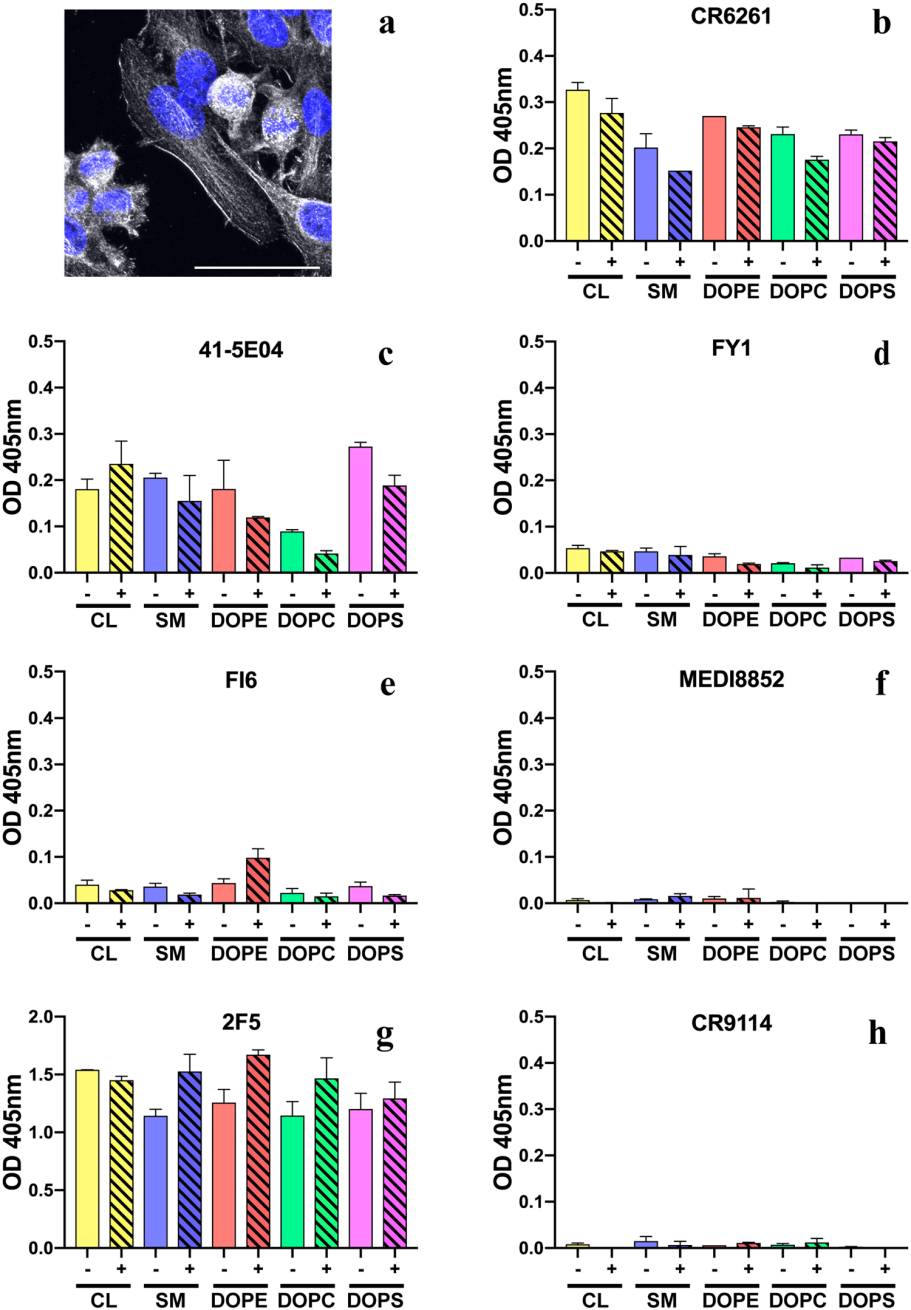
Phospholipid reactivity of HA stem-reactive mAbs. (**a**) Reactivity of CR6261 mAb with the human HEp-2 epithelial cells. To indicate CR6261 (gray scale) reactivity with the plasma membrane maximum intensity Z-projection of 20 stacks was taken with 60x N.A. = 1.2 objective. The cells were co-stained with DAPI (blue). The scale bar is 50 μm. (**b**–**h**) Phospholipid reactivity in enzyme-linked immunosorbent assay (ELISA). Microtiter plates were coated with lipids 1,2-dioleoyl-*sn-*glycero-3-phospho-L-serine (DOPS), 1,2-dioleoyl-sn-glycero-3-phosphocholine (DOPC), 1,2-dioleoyl-sn-glycero-3-phosphoethanolamine (DOPE), cardiolipin (CL) or sphingomyelin (SM), blocked with phosphate-buffered saline (PBS) supplemented with 2% bovine serum albumin (BSA). All mAbs were diluted either in PBS (empty bars, −) or PBS+ 2% BSA (shaded bars, +). Binding was detected with horseradish peroxidase-conjugated secondary Ab (Invitrogen), incubated with ABTS (2,2′-Azinobis [3-ethylbenzothiazoline-6-sulfonic acid]-diammonium salt) and absorbance read at 405 nm. mAb 2F5 was used as control. Reactivity at 0.5 μM is shown as mean ± SD.

We assayed all twelve monoclonal antibodies (mAbs) for polyreactivity by staining cells of a human epithelial line, HEp-2, routinely used to test for antinuclear antibody (ANA) in autoimmune disorders. None of the HA head-directed bnAbs showed HEp-2 staining (Fig. 1b). Of the six stem-directed mAbs, three (FI6v3, CR6261 and 41-5E04) produced significant HEp-2 immunofluorescent staining (Fig. 1b,c). The pattern of FI6 staining was largely cytoplasmic and filamentous, probably from association with intermediate filament components (Fig. 1b), as the antibody did not co-localize with phalloidin stained actin (Fig. S1D). Antibody 41-5E04 stained HEp-2 cells in a pattern very similar to that of FI6 (Fig. 1b). CR6261staining (Fig. 1b), although well above background (Fig. 1c), was less intense than the staining by FI6, 41-5E04, or the autoreactive 564 antibody^22^ (Fig. 1c) but comparable to the control HIV MPER-directed antibody, 2F5 (Fig. 1b,c). CR6261 gave homogeneous cytoplasmic staining with a “fine-speckled” pattern typical of antibodies that bind the Jo-1 histidyl-tRNA synthetase (Figs 1b; S1C)^23^. It also gave noticeable plasma membrane staining (Fig. 2a). The other nine bnAbs did not yield detectable signal (Fig. 1b,c).

We carried out three more specific assays on subsets of the twelve mAbs.

1. Because anti-phospholipid antibodies are present in autoimmune disorders^24–26^ and HIV MPER-directed mAbs 2F5 and 4E10 react with cardiolipin^14^, we tested HA stem-directed mAbs in a lipid-based ELISA (Fig. 2b–h). CR6261 and 41-5E04 (Fig. 2b,c) bound phosphatidylethanolamine (DOPE), phosphatidylserine (DOPS), phosphatidylcholine (DOPC), sphingomyelin (SM) and cardiolipin (CL), to various degrees, although not as strongly as did the control anti-HIV mAb, 2F5 (Fig. 2g). Addition of albumin had no significant effect on the antibody binding to lipids.
2. We screened with a Luminex assay all twelve mAbs with a panel of purified common autoantigens (Table 1; Fig. 3). HA stem-directed CR6261 and FI6 mAbs bound ubiquitin-protein ligase E3A (UBE3A) and the hapten conjugate NP-BSA (Fig. 3). None of the mAbs bound the BSA only control. Consistent with its HEp-2 staining pattern, CR6261 also bound Jo-1. Another HA stem-targeting bnAb, MEDI FY1, showed weak binding with ribonucleoprotein (RNP). C05, the only HA head-directed antibody that reacted with an autoantigen in this assay, bound centromere B.

**Table 1:** Reactivity of anti-influenza HA human bnAbs with autoantigens.

mAb name	IGHV gene	HCDR3 length	Influenza reactivity	HA epitope	HEp-2 cell staining	Luminex AtheNA panel	ProtoArray polyreactivity indices
CR6261	1~69*01	16	group 1	stem	+ cytoplasmic, nuclear	UBE3A, NP-BSA, Jo-1	0.34
FI6	3~30*18	15	group 1 and 2	stem	++ cytoplasmic, nuclear	UBE3A, NP-BSA	−0.09
41-5E04	3~53	20	group 2	stem	++ cytoplasmic, nuclear	−	0.48
CR9114	1~69*06	16	group 1, 2 and B	stem	−	−	0.39
MEDI8852	6~1*01	20	group 1 and 2	stem	−	−	0.02
FY1	6~1*01	20	group 1 and 2	stem	−	RNP	−0.08
CH67	1~2*04	21	group 1	head, RBS	−	−	0.56
CR8033	3~9*01	22	B	head, RBS	−	−	−0.12
6649	4~39*01	19	group 1	head, side	−	−	−0.02
K03.12	1~2*02	28	group 1 and 2	head, RBS	−	−	−0.43
C05	3~23*04	28	group 1 and 2	head, RBS	−	centromere B	0.52
H2526	1~69*01	23	group 1	head, RBS	−	−	0.66

HEp-2 staining: − negative, + positive, ++ bright positive. Luminex AtheNA: − negative, if positive, autoantigen indicated.

**Figure 3:**
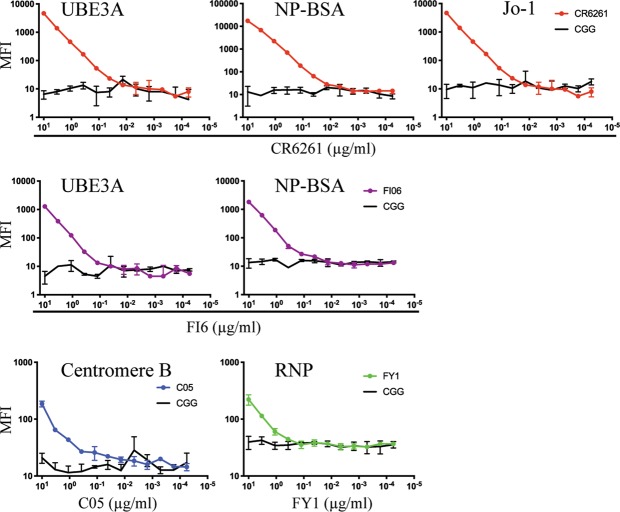
Binding of mAbs CR6261, FI6, C05 and FY1 to autoantigens in Luminex assays. Serial dilutions of mAbs were incubated with the Luminex beads and mean fluorescence intensities (MFI) were read. Mouse IgG1 anti-chick gamma globulin (CGG) was used as control.
3. Our third autoreactivity screen used a ProtoArray microchip^15,16^, a high-content functional protein microarray, which contains over 9,000 human proteins (Fig. 4).

**Figure 4:**
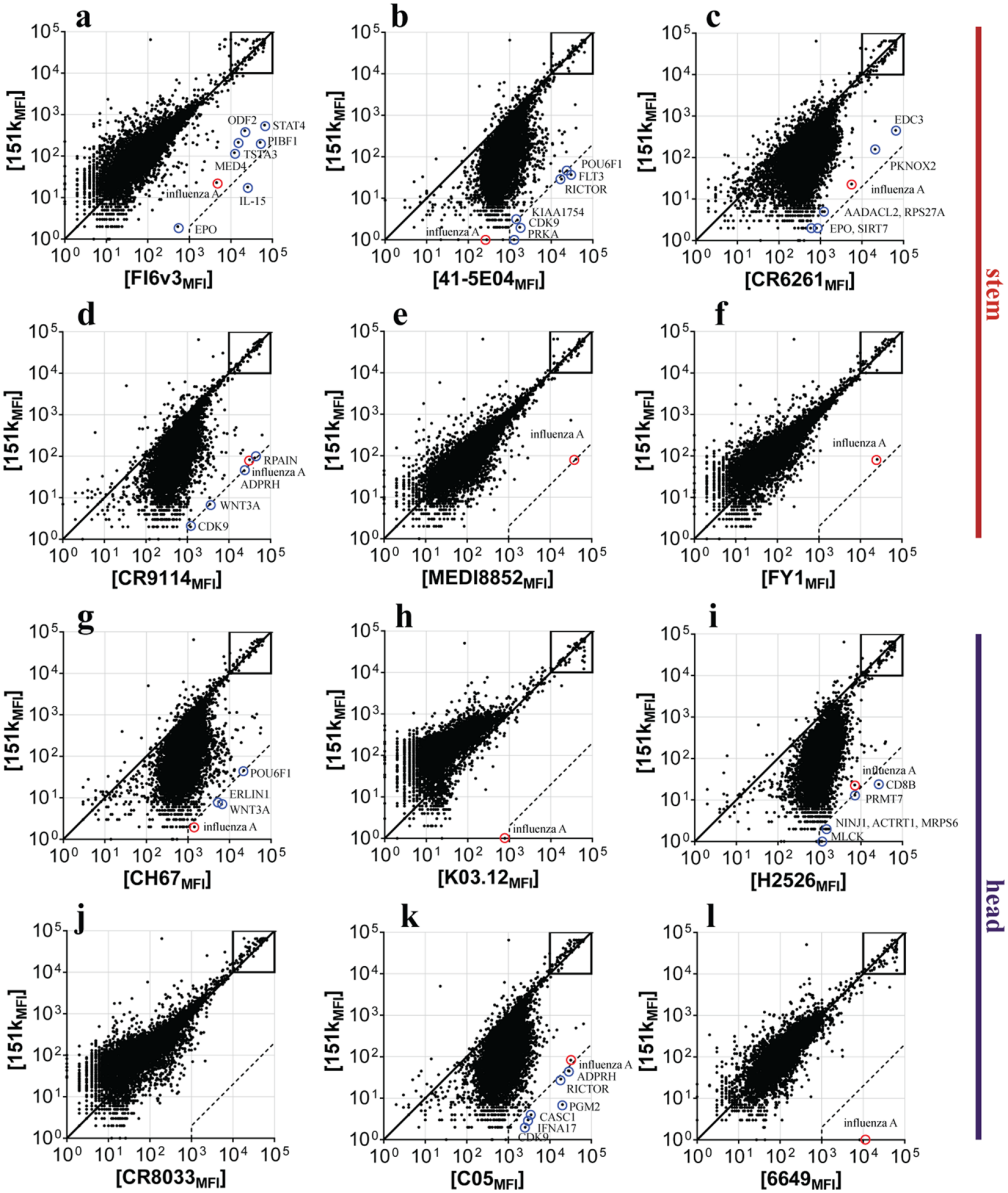
Protein microarray binding of influenza HA bnAbs. (**a**–**l**) Representative ProtoArray summary for protein arrays blotted with (**a**) FI6, (**b**) 41-5E04, (**c**) CR6261, (**d**) CR9114, (**e**) MEDI8852, (**f**) FY1, (**g**) CH67, (**h**) K03.12, (**i**) H2526, (**j**) CR8033, (**k**) C05, (**l**) 6649 or 151 K control. Axis values are relative fluorescence signal intensity (MFI) in the 151 K array (y axis) or test mAb array (x axis). Each dot represents the average of duplicate array proteins. A diagonal line indicates equal binding by test mAb and 151 K. Internal controls for loading of mAb and secondary detection reagent were equally bound by mAb pairs (boxes). Dashed lines indicate the 500-fold signal/background ratio defined as the cutoff for autoreactivity. Circles identify protein autoligands recognized by autoreactive bnAbs.

To assess polyreactivity, the binding patterns of human anti-HA bnAbs were compared to an isotype-matched human myeloma protein 151 K in lot-matched arrays. Essentially, the binding is assessed as a pairwise comparison of signal (anti-HA bnAb) to background (151 K) ratios. As 151 K binds one human protein, betaine-homocysteine methyltransferase 2 (BHMT2)^16^, it is an appropriate negative control for polyreactivity, and serves as a positive control to identify autoreactivity. Previous studies with this assay led us to define the autoreactivity cutoff for HIV bnAbs as a signal/background ratio greater than 500^16^, shown as dashed lines in Fig. 4. Because one of the protein antigens is an influenza A HA, however, we could also define for these tests a more specific cutoff limit. 41-5E04, CR6261 and CR9114 had a highly skewed binding distribution, with some degree of association with most of proteins on the array (Fig. 4b–d). FI6 interacted with interleukin-15 (IL-15) (Fig. 4a; Table 1) and, to a lesser extent, with human erythropoietin (EPO) and progesterone-induced-blocking factor 1 (PIBF1). We also plotted the distance of each ProtoArray data point from the diagonal as a frequency histogram and defined a “polyreactivity index” (PI) as the Gaussian mean of the distances from the reference line. The PIs of CR6261, 41-5E04 and CR9114 were 0.34, 0.48 and 0.39, respectively, indicating a significant displacement of the frequency histogram (Fig. S2). Three HA head-directed bnAbs K03.12, 6649 and CR8033 showed no reactivity in the protein arrays (Figs 4h,k,l and S2), but C05, H2526 (which derives from the V_H_1~69 gene segment) and CH67 bound most of the proteins on the array (Fig. 4k,l,g); Table 1).

In summary, CR6261 and FI6 stem-directed bnAbs scored positively for autoreactivity in the HEp-2 staining, Luminex-based AtheNA, and ProtoArray assays. CR6261 also bound phospholipids. 41-5E04 anti-HA stem mAb was positive in HEp-2, lipid ELISA and ProtoArray assays. FY1, the precursor of an *in vitro* affinity-matured antibody (MEDI8852), bound RNP when tested against the AtheNA panel. The heavy chains of these four antibodies derive from V_H_1~69, 3~30, 3~53 and 6~1, respectively. Another V_H_1~69-derived stem bnAb, CR9114, was polyreactive in the ProtoArray assay. Although none of the six head-directed bnAbs produced HEp-2 nuclear staining, three had polyreactive profiles. C05 had modest autoantigen reactivity in the AtheNA assay, and C05, H2526 and CH67 showed a significant degree of polyreactivity and recognized autoantigens on protein arrays. C05 was isolated by phage display; it is therefore unlikely to have its naturally paired light chain. H2526 has a V_H_1~69 derived heavy-chain variable domain. CH67 does not have any evident characteristics predisposing it to polyreactivity. Although previous work suggested that absence of HEp-2 nuclear staining among the stem antibodies in that study diminished the likelihood of pathogenic polyreactivity^21^, nuclear staining by CR6261, 41-5E04 and FI6 (the latter two with heavy chains not derived from V_H_1~69) and interaction of FI6 and CR6261 with UBE3A as well as CR6261 binding to Jo-1 and of CR6261 and 41-5E04 binding to phospholipids suggest that at least some stem antibodies have pathogenic signatures.

From the autoreactivity profiles of the bnAbs we tested, there appeared to be a correlation between epitope and autoreactivity. Five of the six stem-directed antibodies, CR6261, CR9114, FI6, 41-5E04, and MEDI FY1, were autoreactive to varying degrees. Of these, only CR6261 and CR9114 are encoded by V_H_1~69; the others derive from the V_H_3~30, V_H_3~53 and V_H_6~1 genes, respectively. MEDI8852 showed no detectable autoreactivity, but as this antibody was evolved *in vitro* from MEDI FY1^27^, the introduced mutations may have diminished its autoreactivity profile. Of the six HA head-directed bnAbs, three were polyreactive when assayed on protein arrays, although none were positive in the HEp-2 test. A much larger screen would be needed, however, to conclude that head-directed bnAbs are less prone to autoreactivity than are stem-directed ones.

Influenza HA head epitopes have been under a strong selective immune pressure and have thus been evolving rapidly. Unlike the HA head, the stem has remained largely invariant^28^, probably because its occlusion on virions makes it poorly immunogenic^29^. Anti-HA stem specific antibodies are present in human sera – either natural antibodies or derived from memory - although at low level^30^. Upon (re)exposure to influenza HA in a form that does not occlude the stem (e.g. a split vaccine), one might expect these responses to be rapidly amplified. Yet, in homologous vaccination boost regimens, the immune responses upon re-vaccination are disproportionately skewed towards the globular head epitopes – the head responses increase after re-vaccination, while the stem responses remain the same pre- and post-vaccination^30^. These data are consistent with purging of stem responses through mechanisms of self-tolerance, but a firm conclusion would require a direct test of purging *in vivo*.

Conservation of stem epitopes over decades of influenza virus variation is consistent with the low abundance of stem-directed antibodies and the consequent absence of immune pressure^29^. Imprinting from virus encountered in childhood, which biases subsequent responses to vaccination or infection with a later strain^31^, will then further contribute to the immunodominance of the head. The stem of HA in split vaccines, although largely clustered into rosettes, may be more accessible than on virions, perhaps accounting for the transient appearance of stem-directed antibodies in early recipients of the 2009 vaccine. Suppression by tolerance mechanisms could then explain their failure to persist following subsequent exposure to the same vaccine.

Current research on vaccine strategies for rapidly evolving viral pathogens like HIV and influenza emphasizes epitope-focused, selective clonal induction^32^. For example, recent discussion of a “universal influenza vaccine” has concentrated particularly on HA stem epitopes^33–36^. The data presented here indicate that strategies focused exclusively on the HA stem–or, indeed, on any single epitope–might fail to induce adequate antibody titers owing to negative selection of autoreactive B cell clones. Including multiple conserved epitopes in an epitope-focused vaccine will probably increase the likelihood of a robust response while also decreasing the likelihood that a single mutation will escape an individual’s protective immunity.

## Materials and Methods

### IgG expression and purification

For IgG production the genes for the heavy- and light-chain (kappa or lambda) variable domains were synthesized by Integrated DNA Technologies and cloned into pVRC protein expression vectors containing mouse and human heavy- and light-chain constant domains. Briefly, the bnAb V(D)J sequences were cloned into vectors containing mouse or human IgG1 framework using conventional endonuclease restriction digestion, as previously reported^37^. The mouse isotype was used for HEp-2, lipid ELISA and Luminex screening and the human isotype for ProtoArray microchips. All subsequent secondary reagents were species-specific to limit background signal. Constructs were confirmed by sequencing at the DNA Sequencing Core Facility at Dana Farber Cancer Institute. IgGs were produced by transient transfection of suspension HEK293F cells using polyethylenimine (PEI; Polysciences). Supernatants were harvested 7 days later, clarified by centrifugation and IgGs were purified using Protein G Plus Agarose (Thermo Fisher Scientific): the IgG supernatants were incubated overnight with agarose slurry, eluted with 0.1 M glycine, pH 2.5, neutralized with 1 M Tris-HCl, pH 8.0 and dialyzed against PBS buffer.

### HEp-2 cell staining

Ready to use HEp-20-10 slides (Euroimmun; product No. FA 1512-20) were stained according to manufacturer’s instructions with minor modifications. Recombinant mAbs (mouse IgG1) were diluted to 5 µg/ml or 25 µg/ml in block buffer (0.1% tween, 0.5% BSA in PBS). After one hour of incubation with the mAbs, slides were washed in block buffer and stained with goat anti-mouse IgG-Alexa488 (Life Technologies) and 2 µg/ml DAPI (Sigma) for one hour. After washing, coverslips were mounted with Fluoro-gel (Electron Microscopy Sciences). Images were acquired using an Olympus Fluoview FV1000 confocal system.

### Quantification

Images were analyzed using Cell Profiler^38^. Nuclei were identified by the DAPI signal. Cytoplasmic masks were generated by expanding the nuclei mask by 10 pixels and subtracting the original nucleus mask. Mean fluorescent intensities in the Alexa488 channel were quantified for each cell (defined by nucleus).

### Phospholipid ELISA

Microtiter plates were coated with 5 μg of lipid 1,2-dioleoyl-*sn-*glycero-3-phospho-L-serine (DOPS), 1,2-dioleoyl-sn-glycero-3-phosphocholine (DOPC), 1,2-dioleoyl-sn-glycero-3-phosphoethanolamine (DOPE), cardiolipin (CL) or sphingomyelin (SM), blocked with phosphate-buffered saline (PBS) supplemented with 2% bovine serum albumin (BSA). Recombinant mouse IgG1 mAbs were diluted in PBS or the blocking buffer. Binding was detected with a secondary rabbit anti-mouse IgG coupled with horseradish peroxidase (HRP; Invitrogen), incubated with ABTS (2,2’-Azinobis [3-ethylbenzothiazoline-6-sulfonic acid]-diammonium salt) and absorbance read at 405 nm.

### Luminex binding assay

Luminex assays were performed as reported^39^. In brief, 5 × 10^6^ microspheres (Luminex Corp.) were covalently linked to 25 μg of recombinant protein and incubated with serially diluted mAb (mouse IgG1). Bound mAbs were detected with 4 μg/ml biotinylated goat anti–mouse IgG (SouthernBiotech) (SouthernBiotech), followed by incubation with 5 μg/ml streptavidin-PE (BD). Fluorescence was measured on a Bio-Plex instrument (Bio-Rad).

### ProtoArrays

Recombinant human IgG1 mAbs were screened for binding on protein microarrays (ProtoArray) (catalog no. PAH0525101; Invitrogen) precoated with >9,400 human proteins in duplicate. The binding patterns of human anti-HA bnAbs were compared to the human myeloma protein 151 K in lot-matched arrays. Array-bound anti-human IgG served as the loading control for the detection Ab, and array-bound human IgG served as the loading control for the secondary reagent. Abs were screened for reactive antigens on protein microarrays following the manufacturer’s instructions and as described previously^16^. The ProtoArray microarray (Invitrogen) was blocked and incubated on ice with 2 μg/ml of HA mAb or isotype control 151 K for 90 min. Ab binding to array protein was detected with 1 μg/ml of Alexa Fluor 647-labeled anti-human IgG secondary Ab (Invitrogen). The ProtoArray microarrays were scanned using a GenePix 4000B scanner (Molecular Devices) at 635 nm, with 10-μm resolution. Fluorescence intensities were quantified with GenePix Pro 5.0 program (Molecular Devices) using lot-specific protein location information provided by the microarray manufacturer.

## Supplementary information

Supplementary Information
