# Supplementary material for: Autoreactivity profiles of influenza hemagglutinin broadly neutralizing antibodies
Source: Sci Rep. 2019 Mar 5;9:3492. doi: 10.1038/s41598-019-40175-8 (PMC6401307; doi:10.1038/s41598-019-40175-8)
Supplement: Supplementary file 1 — Supplementary Information [file 41598_2019_40175_MOESM1_ESM.pdf]

Supplementary Information for

Autoreactivity Profiles Of Influenza Hemagglutinin Broadly Neutralizing Antibodies

Goran Bajic[1], Cees E. van der Poel[2], Masayuki Kuraoka[3], Aaron G. Schmidt[4,5], Michael C. Carroll[2], Garnett Kelsoe[3,6] and Stephen C. Harrison[1,7†]

1 Laboratory of Molecular Medicine, Boston Children's Hospital, Harvard Medical School, Boston, Massachusetts 02115 USA
2 Program in Cellular and Molecular Medicine, Boston Children's Hospital, Harvard Medical School, Boston, Massachusetts 02115 USA
3 Department of Immunology, Duke University, Durham, North Carolina 27710, USA
4 Department of Microbiology, Harvard Medical School, Boston, Massachusetts 02115 USA
5 Ragon Institute of MGH, MIT and Harvard, Cambridge, Massachusetts 02139 USA
6 Duke Human Vaccine Institute, Duke University, Durham, North Carolina 27710, USA
7 Howard Hughes Medical Institute, Boston, Massachusetts 02115 USA

[†]Correspondence: harrison@crystal.harvard.edu

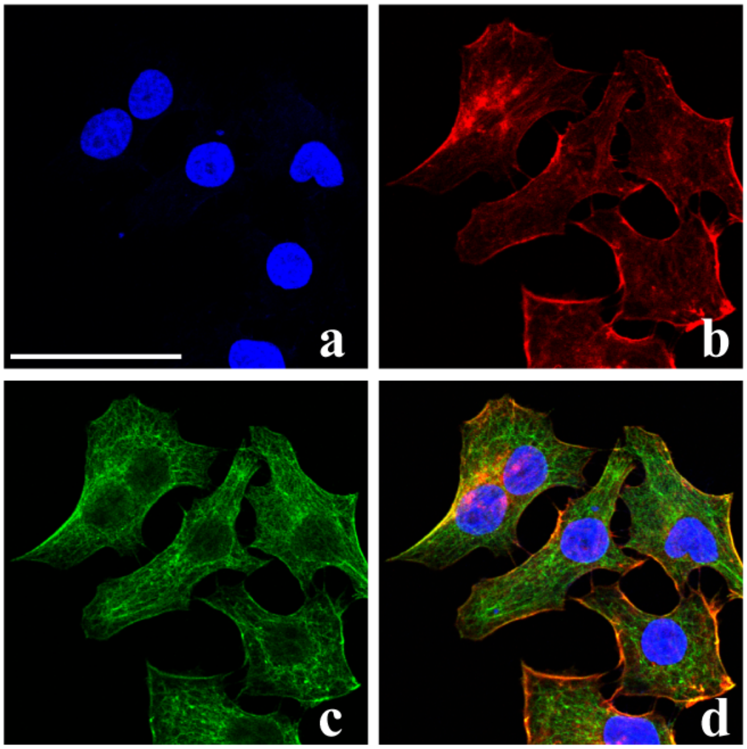

Supplementary Figure 1. Reactivity of HA stem-reactive mAbs FI6 with the human HEp-2 epithelial cells. (**a**) DAPI (blue) (**b**) phalloidin (red) (**c**) FI6 (green) and (**d**) superposition of the three channels. All panels are a single plane taken with 60x objective N.A.=1.2. The scale bar in (A) is 50 µm.

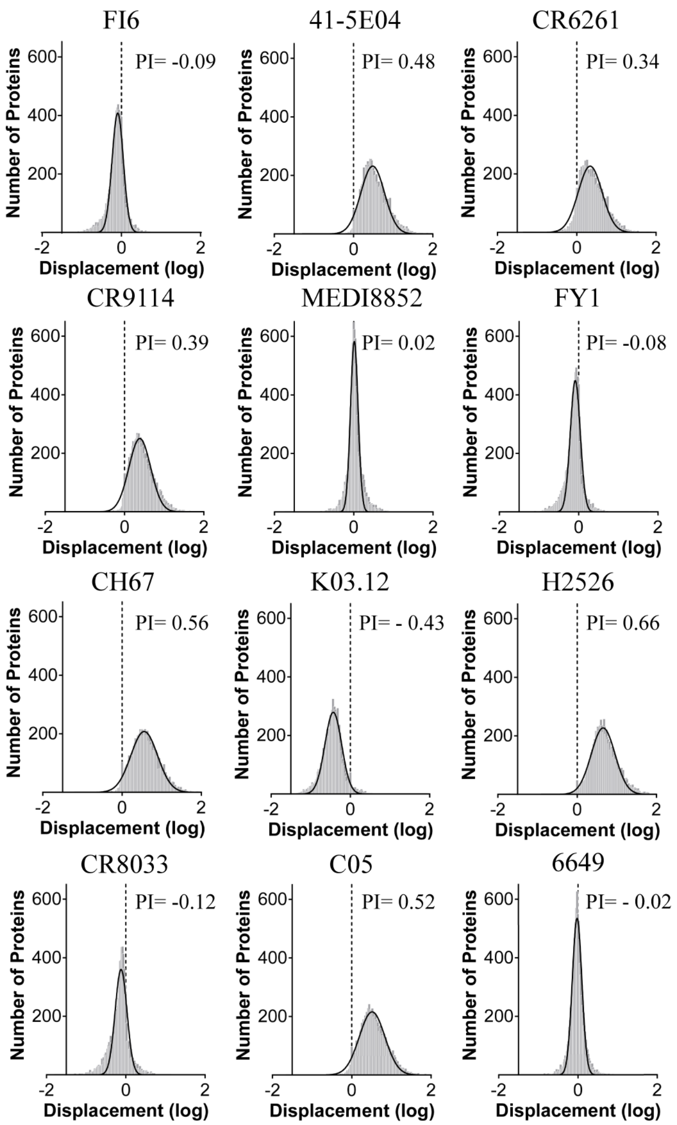

Supplementary Figure 2. Frequency histogram of protein displacement (log) from the diagonal line by mAbs FI6, 41-5E04, CR6261, CR9114, MEDI8852, MEDI FY1, CH67, K03.12, H2526, CR8033, C05 and 6649 compared with the isotype control 151K. The Gaussian mean of protein displacements is termed the polyreactivity index (PI).
